# The Performance of the Super-High-Oleic Acid Safflower (*Carthamus tinctorius*) Oil During Intermittent Frying

**DOI:** 10.3390/foods14050729

**Published:** 2025-02-21

**Authors:** Randy Adjonu, Paul D. Prenzler, Jamie Ayton

**Affiliations:** 1 School of Dentistry and Medical Sciences, Charles Sturt University, Wagga Wagga, NSW 2678, Australia; 2 The Gulbali Institute, Charles Sturt University, Albert Pugsley Place, Wagga Wagga, NSW 2678, Australia; pprenzler@csu.edu.au; 3 School of Agricultural, Environmental and Veterinary Sciences, Charles Sturt University, Wagga Wagga, NSW 2678, Australia; 4 NSW Department of Primary Industries, Wagga Wagga Agriculture Institute, Wagga Wagga, NSW 2650, Australia; jamie.ayton@dpi.nsw.gov.au

**Keywords:** super-high-oleic acid safflower oil, high-oleic acid sunflower oil, canola oil, rice bran oil, frying life, tocopherol, total polar compounds, fatty acid composition, thermo-oxidative stability

## Abstract

High-oleic acid edible oils are appealing, especially for frying, due to their nutritional benefits and high heat stability. This study benchmarked the newly developed super-high-oleic acid safflower oil (SHOSO) against high-oleic acid sunflower (HOSO), conventional canola (CCO), and rice bran (RBO) oils in a frying exercise. The oils were used to fry potato chips for 30 h (90 cycles), and their performance was assessed by measuring the changes in total polar compounds (TPCs), tocopherols, and fatty acid composition. SHOSO contained ~91% oleic acid and had the longest induction time (~35 h) compared with HOSO (~80%; 15.3 h), CCO (~62; 8.8 h), and RBO (~41%; 9.7). After 90 frying cycles, SHOSO’s performance was comparable to that of HOSO, showing the highest increase in TPCs and shortest frying lives (~22.5–25.1 h) compared with CCO (~27.5–33.0 h) and RBO (>30 h). Approximately 97% of the tocopherol in both high-oleic acid oils was α-tocopherol, which was depleted within 6 h. Moreover, SHOSO recorded the largest change in oleic acid, followed by HOSO. SHOSO’s higher oleic acid content influenced its thermal stability and frying life. This study showed SHOSO as a suitable frying oil, and its higher oleic acid content makes it attractive as a functional and healthier fat alternative in food formulations.

## 1. Introduction

The interest in high-oleic acid oils with enhanced heat stability and nutritional properties has increased over the last two decades. High-oleic acid oils have superior frying characteristics, including better heat stability, longer frying life, and improved sensory and nutritional characteristics, to conventional oils [1]. Their high heat stability allows for high-temperature applications, making them attractive oils for commercial food processing and domestic food preparation applications. Furthermore, high-oleic acid oils serve as healthier substitutes for partially hydrogenated oils, which are characterized by elevated levels of trans fatty acids [1]. The utilization of high-oleic acid oils is associated with a reduction in disease risk, improved health outcomes, a diminished public health burden, and enhanced economic benefits for food manufacturers. A 2015 systematic review by Huth et al. [2] reported that replacing high-saturated and -trans fat oils in foods with high-oleic or -linoleic acid oils reduced plasma total and LDL cholesterol, increased HDL cholesterol concentrations, and enhanced cardioprotective effects. High-oleic acid oils exist for canola, peanut, soybean, sunflower [1], and safflower. These typically have oleic acid contents from 73 to 80 in canola, 76 to 82% in peanut, 72 to 84% in soybean, and 80 to 93% in sunflower [1,3,4].

Recently, a super-high-oleic acid safflower variety with an oleic acid content of >90% has been developed in Australia using gene technology for oleochemical applications. Besides a higher oleic acid content, it has a combined linoleic–linolenic acid content of <2% and ~6% saturated fatty acids, making it highly suitable for high-heat applications. In 2018, the Food Standards Australia New Zealand (FSANZ) approved the super-high-oleic acid safflower oil for human food use, while a dietary and risk assessment concluded that the oil was safe for food [5]. Based on Schedule 4 of the FSANZ Code, the super-high-oleic acid oil meets the nutrition content claim requirement due to its higher oleic acid content [5]. The oil’s uniqueness ensures enhanced functionality, stability, biodegradability, and renewability [6].

Frying is a popular food preparation and processing method. Fried food quality depends on several processing factors, including the oil type and composition, food type, fryer type, and frying conditions. These include the frying temperature, frying time, frequency of frying, number of frying cycles, quantity of food and oil used, and oil replenishment. The oil type and composition (fatty acid composition and antioxidant compounds) are independent determinants of fried foods’ physicochemical and sensory properties, safety, shelf life, and nutritional quality. Moreover, the fatty acid type and content influence an oil’s frying life, with frying life being correlated with increasing saturated fatty acid and oleic acid and decreasing polyunsaturated fatty acid contents, such as linoleic and linolenic acid contents [3]. High-linoleic and -linolenic acid oils are prone to oxidation and polymerization, generating potentially toxic compounds, such as polycyclic aromatic hydrocarbons, acrylamide, heterocyclic amines, aldehydes, etc. [7], that are detrimental to human health. Hence, they are less suitable for high-temperature applications, making the oil type one of the most critical determinants of fried food quality.

Frying trials are a cost-effective way to compare cooking oils’ performance and frying life. Frying trials are often conducted with common foods, such as potato chips or chicken nuggets, and parameters, including the total polar compound and free fatty acid contents, changes in fatty acid composition, and loss of antioxidants, are measured to determine an oil’s frying life [3,8]. Frying exercises can be continuous or intermittent. This study compared the frying life of the newly developed super-high-oleic acid safflower oil with commonly used frying oils—high-oleic acid sunflower (~81% oleic acid), conventional canola (~62% oleic acid), and rice bran (~41% oleic acid) oils—in an intermittent frying exercise. This is the first report on the food application of super-high-oleic acid safflower oil, and it provides fundamental scientific data about its food-processing applications.

## 2. Materials and Methods

### 2.1. Materials

Super-high-oleic acid safflower oil (SHOSO) was refined and supplied by Plenty Foods Pty (Kingaroy, QLD, Australia). Plenty Foods also supplied high-oleic acid sunflower oil (HOSO). Conventional canola oil (CCO) was obtained from an Australian processor, and rice bran oil (RBO) and potato chips (10 mm straight-cut chips [par-fried], HARVEST CHOICE) were purchased from PFD Food Services Pty Ltd. (Wagga Wagga, NSW, Australia). Fatty acid methyl acid ester (FAME) standard was purchased from Merk Life Science Pty Ltd. (Bayswater, VIC, Australia). All reagents were of analytical grade.

### 2.2. Assessment of Fresh Oil Quality

The fresh oils were tested for their free fatty acid (FFA) content (ISO, 2009, Method ISO660:2009) [9], peroxide value (PV) (American Oil Chemists’ Society [AOCS] Method Cd 8b-90) [10], and induction time by Rancimat (AOCS Method Cd 12b-92) [10]. Testing was conducted at the NSW Department of Primary Industries Oil Testing Laboratory (Wagga Wagga, Australia).

### 2.3. Intermittent Frying Exercise

Frying was conducted in a 2 × 5 L stainless-steel double-pan deep fryer (Model FFA2002, Anvil Axis, Roodepoort, South Africa), as previously described in [11], with slight modifications. Briefly, 0.5 kg of frozen potato chips was fried in 5 kg of oil at 180 ± 5 °C. Frying was completed every 20 min for 6 h on day 1 and 8 h on days 2 to 4 (a total of 90 cycles). The frying oil was filtered on days 2, 3, and 4 to remove food residue. Frying was carried out without oil replenishment, and the amount of chips was adjusted to the oil level on days 2, 3, and 4 to maintain an oil-to-food ratio of approximately 10:1 (*w*:*w*). The oil-to-food ratio was determined based on prior research conducted by our team [11]. Given that the oils were not replenished during the frying process, this ratio was established to ensure that the oil was sufficient to complete the frying exercise effectively.

### 2.4. Determination of Fatty Acid Composition

The fatty acid composition was determined as methyl esters following the International Olive Council (IOC) standard method, COI/T.20/Doc No. 33/Rev. 1 [12]. The fatty acids were trans-esterified into fatty acid methyl esters (FAMEs) using methanolic potassium hydroxide. The FAMEs were analyzed by capillary gas chromatography using an Agilent 7890 Gas Chromatograph (Agilent Technologies, Mulgrave, VIC, Australia) fitted with a BPX70 GC column (30 m × 0.22 mm × 0.25 µm) (SGE Analytical Science Pty Ltd., Ringwood, VIC, Australia) and a flame ionization detector (FID) detector. The chromatographic conditions are described in the IOC standard method COI/T.20/Doc No. 33/Rev. 1.

### 2.5. Tocopherol Analysis

Tocopherol was quantified by high-performance liquid chromatography (HPLC) according to the International Organization for Standardization Method ISO9936:2016 [13]. The oil samples were dissolved in heptane and separated with an Agilent 1290 HPLC (Agilent Technologies, Mulgrave, VIC, Australia) using a silica column (250 mm × 4.6 mm) and UV detection at 292 nm. The tocopherols were quantified using the external calibration curves of alpha and gamma tocopherols.

### 2.6. Determination of Oil Frying Life

Oil frying life was determined by measuring the total polar compound (TPC) content using a Testo 270 oil tester (Testo Pty Ltd., Croydon South, VIC, Australia) at 150 °C per the manufacturer’s instructions. For the TPC readings, each frying pan was visually divided into a lower and upper section, and a reading was taken from each section, with the average used as the TPC value. Each day, the TPC was measured after each frying cycle (i.e., every 20 min for 6 h on day 1 and 8 h on days 2 to 4) for a total of 90 cycles over the 4 days of frying.

### 2.7. Statistical Analysis

The frying experiments and oil quality analysis were performed in duplicate, and the means ± standard deviations were reported.

## 3. Results

### 3.1. Pre-Frying Oxidation States of Oils

Table 1 shows the pre-frying quality data of the oils. Canola oil and SHOSO had the lowest peroxide values (<2 mEq O_2_/kg oil), while RBO recorded the highest value. The induction time of SHOSO was ~2.3 times that of the HOSO and ~3.6 to 4 times that of RBO and CCO (Table 1).

### 3.2. Changes in Free Fatty Acid (FFA) Content During Frying

**Figure 1:**
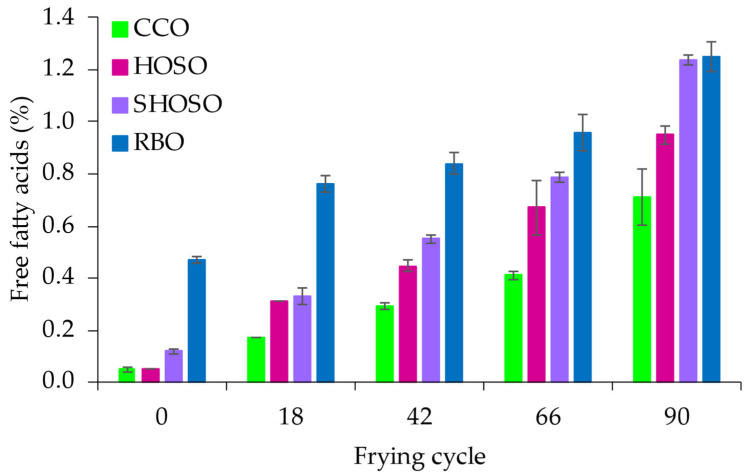
Changes in free fatty acid content during frying of potato chips in canola (CCO), high-oleic acid sunflower (HOSO), super-high-oleic acid safflower (SHOSO), and rice bran (RBO) oils. Frying was conducted for 30 h, equivalent to 90 cycles.

The FFA content in the fresh oils was between 0.05% and 0.47%. High-oleic acid sunflower oil and CCO had the lowest initial FFA contents (0.05%), followed by SHOSO (0.12%), while RBO recorded the highest FFA value (0.47%). During frying, the FFA contents of all the oils increased progressively with the number of frying cycles (Figure 1). After 90 frying cycles, SHOSO and RBO showed the highest FFA contents (approximately 1.2%), while CCO recorded the lowest (approximately 0.7%). Also, HOSO and SHOSO recorded the largest absolute changes in FFA content, at 0.90 and 1.12, respectively, compared with 0.66 and 0.78 for CCO and RBO.

### 3.3. Changes in Fatty Acid Composition (FAC) During Frying

The FACs for the fresh oils are given in Table 2 (D0). Super-high-oleic acid safflower oil contained the highest monounsaturated fatty acid (MUFA) content, primarily as oleic acid (C18:1), followed by HOSO, CCO, and RBO. The opposite was true for the saturated fatty acid (SFA) content, which had palmitic acid (C16:0) as the primary SFA. Linoleic acid (C18:2) was the main polyunsaturated fatty acid (PUFA), highest in RBO and lowest in the SHOSO. Canola oil contained the highest linolenic acid content, while the two high-oleic acid oils were nearly devoid of linolenic acid. The initial trans fatty acid content was low in all oils (Table 1), with HOSO recording the lowest TFA content (0.11%) and then SHOSO (0.34%), RBO (0.49%), and CCO (0.62%).

**Figure 2:**
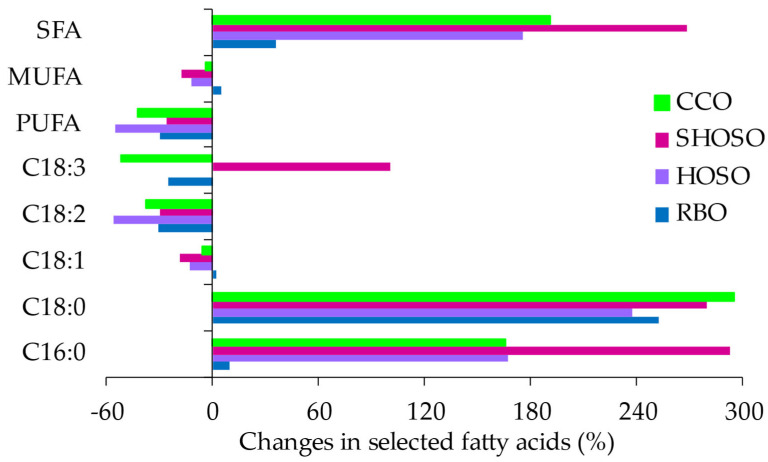
Changes in the major fatty acid compositions of oils after four days of frying potato chips in canola (CCO), high-oleic acid sunflower (HOSO), super-high-oleic acid safflower (SHOSO), and rice bran (RBO) oils. Frying was conducted for 30 h, equivalent to 90 cycles.

Table 2 (D4) also shows the changes in FAC at the end of frying on day 4. For all oils, the SFAs palmitic and stearic (C18:0) acids increased, whereas linoleic and linolenic acids decreased. The oleic acid content decreased for all oils except for RBO. The changes in the major fatty acids were consistent with the changes in the MUFAs, PUFAs, and SFAs. Canola oil had the largest change in PUFA content at the end of frying day 4, consistent with its high linolenic acid content (Figure 2). Conversely, SHOSO showed the least change in PUFAs, consistent with the very low PUFA content, at ~1.9% (Figure 2).

### 3.4. Changes in Tocopherols During Frying

Fresh CCO contained the highest total tocopherol content, and RBO contained the least (Table 3; D0). The super-high-oleic acid safflower oil’s tocopherol content was about half (~0.55) that of the HOSO. In CCO, γ-tocopherol constituted ~64.1%, with a gamma–alpha-tocopherol ratio of ~1.8. Conversely, ~97.4% of the high-oleic acid oils and 71.1% of RBO’s tocopherol content was the alpha isomer, with gamma–alpha-tocopherol ratios of ~0.3 and ~0.4, respectively. Table 3 also shows the tocopherol retention during frying. At the end of day 1, the two high-oleic acid oils were depleted of tocopherols (<8 mg/kg oil), but CCO and RBO had ~76.1% (70.9% alpha and 70.2% gamma) and 70.4% (77.7% alpha and 72.2% gamma) remaining, respectively. Canola oil’s tocopherol content was depleted by the end of day 4, but RBO had ~68 mg/kg oil (27.6%; 33.4% alpha and 13.3% gamma) remaining.

### 3.5. Changes in Total Polar Compounds (TPCs) During Frying

**Figure 3:**
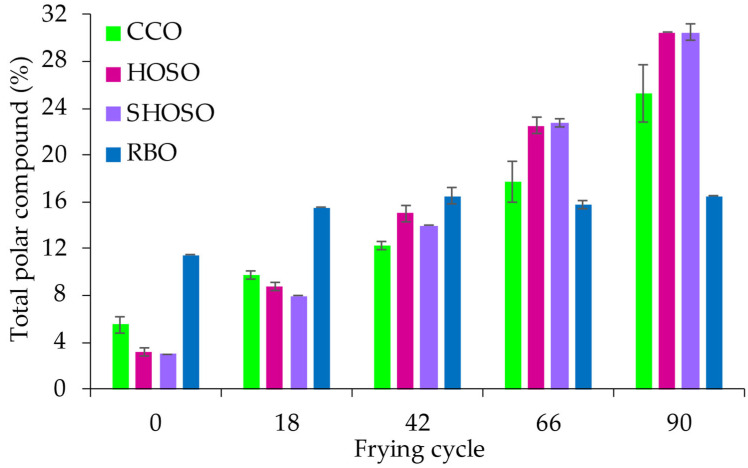
Changes in total polar compound contents during frying of potato chips in canola (CCO), high-oleic acid sunflower (HOSO), super-high-oleic acid safflower (SHOSO), and rice bran (RBO) oils. Frying was conducted for 30 h, equivalent to 90 cycles.

Prior to frying, SHOSO and HOSO contained the least TPCs (3.0 ± 0.0 and 3.3 ± 0.4, respectively), followed by CCO (5.5 ± 0.7%) and RBO (11.5 ± 0.0%), which contained the highest TPC content. During frying, the SHOSO and HOSO recorded the highest TPC values and had a similar TPC evolution. Although RBO had the highest initial TPC content, it showed the least increase in TPCs, with TPCs remaining below 17% after 90 frying cycles. Notably, RBO had the least absolute change in TPCs (5%) compared with the other oils, CCO (19.8%), HOSO (27.3%), and SHOSO (27.5%) (Figure 3).

## 4. Discussion

### 4.1. Pre-Frying Oil Composition and Quality

Super-high-oleic acid safflower oil and CCO had the lowest initial peroxide values as these had been freshly refined and sourced directly from local processors. Conversely, the PVs of HOSO and RBO indicated increased oxidation. Both oils were imported products; hence, longer transit times and handling during packaging and distribution may have led to increased oxidation. Canola and HOSO had low initial FFA contents, which were typical of these oils when adequately refined and were concordant with previous reports [14,15]. The slightly higher FFA content of SHOSO oil reflects the refinery process’s capacity to reduce the FFA content during oil deodorization. Regardless, SHOSO oil’s FFA content was consistent with that reported for conventional low-oleic acid/high-linoleic acid safflower oils [16]. The RBO’s FFA content was also consistent with reported data, ranging between 0.29 and 0.55% [17].

The FACs were typical of the oils [11,18,19], with RBO displaying a more balanced and robust FAC, with a SFA, MUFA, and PUFA ratio of 1:1.3:0.7. A balanced FAC enhances an oil’s heat stability and nutritional functionalities. Moreover, high oleic acid and SFA contents increase an oil’s heat stability as they are less prone to polymerization and oxidation reactions compared with linoleic and linolenic acids [20]. Hence, safflower oil, with its super high oleic acid content, should display increased thermo-oxidative stability compared with CCO and HOSO. Besides FAC, factors including the tocopherol content, refined oil’s quality and oxidation state, age of the oil, and food type affect an oil’s heat stability and frying life. Nutritionally, high-MUFA diets may contribute to reduced fasting plasma glucose, blood pressure, and body weight and increased HDL cholesterol [21]. Thus, SHOSO is a promising nutritionally functional oil in food formulations and warrants further research.

In this study, HOSO and CCO had relatively high tocopherol contents, while SHOSO had a moderate tocopherol content, and RBO had the lowest. Canola oil contained an almost balanced ratio of the alpha and gamma forms, whereas HOSO, SHOSO, and RBO contained mainly the alpha form. The α-tocopherol content of super-high-oleic acid safflower oil was ~55% that of HOSO and about twice (~178%) that of RBO. Conversely, the α-tocopherol content in RBO constituted ~31% of that in HOSO. Both tocopherol forms are effective antioxidants in oils; however, they demonstrate distinct antioxidant activities in food systems [22]. Compared with SHOSO and HOSO, the abundance and diversity of tocopherol isomers in CCO can enhance its thermo-oxidative stability during food-processing operations. Besides tocopherols, RBO contains tocotrienol (~188–395 mg/kg) and gamma-oryzanol (>3000 mg/kg) [23,24]. These vitamin E variants and gamma-oryzanol may exert a beneficial synergistic effect on RBO’s heat stability and functionality.

A longer induction time and higher oleic acid content correlate with greater oil auto-oxidation stability. Thus, safflower oil’s super high oleic acid content and moderate tocopherol content accounted for its long induction time. Although CCO and RBO recorded the shortest induction times, these were higher than the values reported by Maszewska et al. [25].

### 4.2. Effects of Frying on Oil Composition and Frying Life

The repeated frying of oils causes thermo-oxidative and hydrolytic degradation of triglycerides and the polymerization of fatty acids, increasing an oil’s polar compound content. Therefore, measuring the total polar compound (TPC) content is a reasonable assessment of deteriorative changes in frying oils and allows the estimation of an oil’s frying life [26]. Stable oils show less change or increase in their TPC content during frying. In Figure 2, the TPCs of oils increased with frying time, particularly for SHOSO and HOSO. Several countries have set a TPC discard limit of 24–27% during frying [26]. In this study, SHOSO and HOSO recorded the highest TPC values, hence the shortest frying lives (~22.5–25.1 h), while CCO had a moderate frying life (~27.5–33.0 h) (Figure 2). Rice bran had the longest frying life, with a TPC content below 24% after >38 h of frying. Super-high-oleic acid safflower oil and HOSO achieved ~66–75 frying cycles compared with CCO at 82–99 cycles and RBO at >114 cycles. Overall, the TPC changes in the high-oleic acid oils were consistent with previous reports on high-oleic acid oils [3].

Studies have reported better heat stability and longer frying lives for high-oleic acid oils, which are not consistent with the current study. For example, Li et al. [27] reported lower TPC values for high-oleic acid sunflower oil than canola and cottonseed oils when used to fry French fries but had similar TPCs when used to fry chicken and fish nuggets. In a rotational frying exercise, high-oleic acid canola oil had a lower polar compound content and showed increased heat stability compared with conventional canola and soybean oils [28]. Discrepancies between the current and previous studies may be due to, firstly, differences in frying protocols, including the oil-to-food ratio, fryer and food type, frying temperature, frying cycles, oil replenishment, pre-frying oil quality, and rotational versus static frying. Li et al. [27] used a food–oil ratio of 1:50, and Przybylski et al. [28] used a ratio of 1:8.75, while the current study used a 1:10 ratio. Moreover, Przybylski et al. [28] replenished the frying oil with fresh oil every second day, extending the oil’s frying life and the number of cycles and influencing the degradation reactions during frying. These factors may limit the generalization of the current data as SHOSO may behave differently when used to fry different foods and in other industrial applications. Secondly, studies have usually compared high-oleic acid oils against their regular types [28], providing direct evidence of the effect of oleic acid modification on frying performance as opposed to the cross-commodity comparison in the current study.

Consistent with the TPC content, SHOSO and HOSO had the largest absolute change in FFAs. Free fatty acids contribute to an oil’s polar compound content. This observation is consistent with the greater triglyceride degradation in the super-high/high-oleic acid oils compared with CCO and RBO. Although comparing frying data from different studies is imprecise due to differences in frying protocols, the current FFA values were consistent with previous data on repeatedly fried oils [28,29]. Overall, RBO had the least change in FAC, indicating better frying stability, consistent with the TPC and FFA data (Figure 1 and Figure 3), hence the longest frying life.

Compared with the other oils, SHOSO had a combined linoleic and linolenic acid content of <2.0% and the least change in these fatty acids, indicating that the PUFAs played a limited to no role in the thermo-oxidative stability of this oil. However, SHOSO had the largest decrease in MUFAs as oleic acid, implicating oleic acid as the driver of thermo-oxidative stability. In response to changes in MUFAs, SHOSO also had the largest SFA increase (Figure 2). Compared with MUFAs, SFAs are stable to heat degradative reactions, and the relative increases are only in response to the degradation of the unsaturated fatty acids. For HOSO, oleic and linoleic acids were drivers of stability, while oleic, linoleic, and linolenic acids influenced CCO’s stability. For RBO, both SFAs (~26.2%) and MUFAs, principally as oleic acid (~1.9%), increased at the end of frying day 4, highlighting the heat stability of these fatty acids in RBO. Consistent with previous studies, Latha and Nasirullah [30] also reported a marginal increase in RBO’s oleic acid (~1.8%) when heated at 180 °C for 8 h. Thus, in RBO, linoleic acid, the major PUFA (with a loss of approx. 10.1) influenced, to a greater extent, the oil’s thermo-oxidative stability during frying.

In the current study, although SHOSO and HOSO contained higher oleic acid contents (Table 2), they were depleted of tocopherols (mainly α-tocopherol) within 6 h of frying (Table 3), indicating a limited antioxidative protective capacity from tocopherols. Thus, after the first 6 h, SHOSO and HOSO’s heat stability, and, hence, their frying lives, were likely governed by their higher oleic acid contents. Conversely, CCO retained ~193 mg/kg of tocopherols at the end of day 3, whereas only RBO retained both α- and γ-tocopherols (~68 mg/kg total) at the end of frying day 4 (Table 3). Tocopherols contributed to their oxidative stabilities and their longer frying lives (Figure 3). Besides tocopherols, RBO contains tocotrienols and γ-oryzanol [23,24], which may exert a protective effect on tocopherols and act synergistically to enhance rice bran oil’s heat stability [18].

## 5. Conclusions

The newly developed super-high-oleic acid safflower oil was compared with high-oleic acid sunflower, canola, and rice bran oils in a frying exercise. Super-high-oleic acid safflower oil contained ~91% oleic acid and a combined linoleic and linolenic acid content of <2% and had the longest induction time compared with high-oleic acid sunflower, canola, and rice bran oils. After 90 frying cycles, the super-high-oleic acid safflower oil’s frying life was comparable to that of high-oleic acid sunflower oil but shorter than that of canola and rice bran oils. Both super-high-oleic acid safflower and high-oleic acid sunflower oils’ tocopherols were depleted within 6 h of frying. This may have led to their shorter frying lives compared with the canola and rice bran oils. Overall, the super-high-oleic acid safflower oil’s frying life seems to be influenced by both its higher oleic acid and moderate tocopherol contents and warrants further investigation.

## Figures and Tables

**Table 1:** Pre-frying quality data of canola, high-oleic acid sunflower, super-high-oleic acid safflower, and rice bran oils.

Oil Type	Free Fatty Acids (% Oleic Acid)	Peroxide Value (mEq O_2_/kg Oil)	Induction Time (h)	Trans Fatty Acid (%)
CCO	0.05 ± 0.01	<2	8.8 ± 0.5	0.62 ± 0.01
HOSO	0.05 ± 0.00	5.0 ± 0.0	15.3 ± 0.9	0.11 ± 0.01
SHOSO	0.12 ± 0.01	<2	35.1 ± 0.5	0.34 ± 0.01
RBO	0.47 ± 0.01	7.0 ± 0.0	9.7 ± 0.5	0.49 ± 0.00

NB: CCO = canola oil, HOSO = high-oleic acid sunflower oil, SHOSO = super-high-oleic acid safflower oil, and RBO = rice bran oil.

**Table 2:** Fatty acid compositions of fresh oils and after four days of frying.

Oil Type	Day	C16:0	C18:0	C18:1	C18:2	C18:3	MUFAs	PUFAs	SFAs
CCO	D0	4.3 ± 0.0	2.1 ± 0.0	61.9 ± 0.1	19.0 ± 0.0	9.9 ± 0.0	63.5 ± 0.0	28.9 ± 0.1	7.7 ± 0.1
	D4	11.4 ± 0.1	8.3 ± 0.1	58.5 ± 0.2	11.9 ± 0.4	4.8 ± 0.3	61.0 ± 0.2	16.7 ± 0.6	22.4 ± 0.4
HOSO	D0	4.5 ± 0.0	2.7 ± 0.0	80.3 ± 0.1	10.3 ± 0.0	0.2 ± 0.0	80.8 ± 0.0	10.5 ± 0.1	8.8 ± 0.1
	D4	12.0 ± 0.1	9.1 ± 0.0	69.5 ± 0.1	4.5 ± 0.1	0.2 ± 0.0	71.2 ± 0.1	4.7 ± 0.1	24.2 ± 0.1
SHOSO	D0	2.8 ± 0.0	2.4 ± 0.0	90.9 ± 0.0	1.7 ± 0.0	0.1 ± 0.0	91.9 ± 0.0	1.9 ± 0.0	6.2 ± 0.0
	D4	11.0 ± 0.1	9.1 ± 0.1	73.8 ± 0.1	1.2 ± 0.0	0.2 ± 0.0	75.8 ± 0.1	1.4 ± 0.0	22.8 ± 0.1
RBO	D0	19.9 ± 0.1	2.1 ± 0.0	41.0 ± 0.1	33.1 ± 0.1	1.2 ± 0.0	41.8 ± 0.1	34.3 ± 0.1	24.0 ± 0.0
	D4	21.8 ± 0.1	7.4 ± 0.1	41.8 ± 0.1	23.0 ± 0.4	0.9 ± 0.0	43.7 ± 0.1	23.9 ± 0.4	32.5 ± 0.2

NB: CCO = canola oil, HOSO = high-oleic acid sunflower oil, SHOSO = super-high-oleic acid safflower oil, and RBO = rice bran oil; C16:0 = palmitic acid, C18:0 = stearic acid, C18:1 = oleic acid, C18:2 = linoleic acid, C18:3 = linolenic acid, MUFAs = monounsaturated fatty acids, PUFAs = polyunsaturated fatty acids, and SFAs = saturated fatty acids.

**Table 3:** Tocopherol concentration (mg/kg oil) in fresh and fried oils.

Day	Tocopherol	CCO	HOSO	SHOSO	RBO
D0	Alpha	269	570	311	175
	Gamma	480	15	9	71
	Total	749	584	320	246
D1	Alpha	191	<8	<8	136
	Gamma	337	<8	<8	51
	Total	528	<8	<8	187
D2	Alpha	67	<8	<8	100
	Gamma	125	<8	<8	36
	Total	193	<8	<8	136
D4	Alpha	<8	<8	<8	59
	Gamm	<8	<8	<8	9
	Total	<8	<8	<8	68

NB: CCO = canola oil, HOSO = high-oleic acid sunflower oil, SHOSO = super-high-oleic acid safflower oil, and RBO = rice bran oil.

## Data Availability

The original contributions presented in the study are included in the article, further inquiries can be directed to the corresponding author.
